# Spatial distribution of pregnancy and early motherhood among late adolescent girls in Ethiopia using data from the Ethiopia Demographics and Health Survey 2019: Spatial and multilevel analyses

**DOI:** 10.1371/journal.pone.0306170

**Published:** 2024-08-01

**Authors:** Nega Tezera Assimamaw, Tewodros Getaneh Alemu, Masresha Asmare Techane, Chalachew Adugna Wubneh, Getaneh Mulualem Belay, Tadesse Tarik Tamir, Addis Bilal Muhye, Destaye Guadie Kassie, Amare Wondim, Bewuketu Terefe, Bethelihem Tigabu Tarekegn, Mohammed Seid Ali, Beletech Fentie, Almaz Tefera Gonete, Berhan Tekeba, Selam Fisiha Kassa, Bogale Kassahun Desta, Amare Demsie Ayele, Melkamu Tilahun Dessie, Kendalem Asmare Atalell

**Affiliations:** 1 Department of Pediatrics and Child Health Nursing, School of Nursing, College of Medicine and Health Sciences, University of Gondar, Gondar, Ethiopia; 2 School of Nursing, College of Medicine and Health Sciences, University of Gondar, Gondar, Ethiopia; PLOS: Public Library of Science, UNITED KINGDOM

## Abstract

**Background:**

Various governmental and non-governmental organizations in Ethiopia are striving to decrease adolescent pregnancy by enacting laws against early marriage, developing a national youth and adolescent reproductive health strategy, legalizing abortion, and developing an HIV/AIDS policy for youth; however, the issue of teenage pregnancy& early motherhood remains a major concern.

**Methods:**

Data were obtained from the Ethiopian Demographics and Health Survey (EDHS) in 2019. A total sample of 2210 adolescents was included in our study. Spatial autocorrelation, hotspot analysis, and spatial interpolation were used to observe significant spatial variation and clustering and to predict the prevalence of pregnancy in an unsampled area among adolescent girls in Ethiopia; a multilevel binary logistic regression model was fitted to identify factors associated with the outcome variable. The adjusted odds ratio was calculated with a 95% confidence interval, and the variables with a p-value 0.05 in the multivariable multilevel logistic regression were determined to be statistically significant.

**Results:**

Global spatial autocorrelation analyses showed that the spatial distribution of late-adolescent pregnancy and early motherhood varied across Ethiopia (the Global Moran’s Index I value showed GMI = 0.014, P 0.001). The spatial distribution revealed a high cluster (hot spot) of late-adolescent pregnancy and early motherhood in most parts of Gambella, Afar, Benishangul-Gumuz, the eastern part of Oromia, and Somalia. In the multivariable multilevel analysis, being 17 years old (AOR = 3.43; 95% CI: 1.54–7.59), 18 years old (AOR = 14.92; 95% CI: 6.78–32.8), and 19 years old (AOR = 8.44; 95% CI: 4.06, 17.56), married (AOR = 25.38; 95% CI: 15.33, 42.02), having completed primary, secondary, and higher education (AOR = 0.45; 95% CI: 0.21–0.95), and being at Gambela (AOR = 3.64; 95% CI: 1.04, 12.75) were significant predictors of late adolescent pregnancy and early motherhood.

**Conclusion:**

Overall, the prevalence of late-adolescent pregnancy and early motherhood was found to be high. At the individual level, marital status, educational attainment, and age of adolescents were significant predictors of pregnancy and early motherhood, and regions were found at a community level associated with pregnancy and early motherhood among late adolescents. Therefore, late-adolescent girls should be educated about menstruation, sexual intercourse, pregnancy, and contraceptives before they reach early adolescence.

## Introduction

The World Health Organization (WHO) defines late adolescence as ranging from 15 to 19 years of age [1]. Adolescents undergo significant physical, cognitive, emotional, and behavioral changes as they gain autonomy. The use of drugs and alcohol, smoking, and engaging in sexual activity can all lead to sexual and reproductive health problems, such as unwanted pregnancies and sexually transmitted illnesses (STIs) [2].

Although adolescent pregnancies are a global issue affecting both developing and developed countries, they are more likely to occur in underprivileged communities [3]. Worldwide, the adolescent fertility rate has declined by 11.6% in 20 years. However, the rates vary significantly by region. East Asia and Central Africa have the largest disparity, each with 7.1 and 129.5 [4].

Girls in the least developed countries marry at least 39% before they turn 18; and 12% before they turn 15 [5]. Each year, roughly 21 million females between the ages of 15 and 19 become pregnant, 12 million give birth, and at least 777,000 girls under 15 give birth, mainly in low- and middle-income countries [6,7]. Moreover, approximately 10 million unplanned pregnancies occur each year among teenagers in these areas[7].

Globally, the rate of teenage pregnancy varies greatly; the highest rates are observed in Bangladesh (43%), Nepal (11.1% to 47.3%), and Jordan (25%). Some nations, on the other hand, have the lowest rates of teenage pregnancy worldwide: North Korea, with 0.5608 births per 1,000 people, South Korea, with 1.6584; other low-rate nations include 3.0578 in Switzerland, 3.2662 in Hong Kong, and 3.8334 in Singapore [8].

The countries of Africa had the highest rates of teenage pregnancies worldwide: 203.604 births per 100,000 people in Niger; 175.4438 in Mali; 166.6028 in Angola; 142.5334 in Mozambique; 141.6722 in Guinea; 137.173 in Chad; 136.972 in Malawi; and 135.464 in Cote d’Ivoire [9]. The percentage of pregnancies among teenagers is 13%, according to the EDHS 2016 Findings Report [10]. Additionally, there is a difference in residence: 15% live in rural areas and 5% in metropolitan areas. Pregnancies in Ethiopia are distributed unevenly, with 23% in Afar, 8% in Amhara, and 3% in Addis-Ababa [10].

Studies have shown that many factors contribute to the inappropriate and inconsistent use of contraceptives, including misconceptions, lack of knowledge, social norms condemning premarital sex, contraception, early marriage, lack of education funding, drug abuse, low education attainment, lack of privacy at clinics, and lack of agency [11–14].

Young girls have been abused by societies by being forced to marry and have children at an early age, which is harmful to them, their families, and society [15–18]. Adolescent girls (15–19 years) are most likely to die from complications during pregnancy and childbirth, with LMICs accounting for 99% of all maternal deaths. [19]. There are 5.6 million abortions every year among girls aged 15 to 19, of which 3.9 million are unsafe [7]. The risk of eclampsia, puerperal endometritis, and systemic infections is higher among adolescent mothers (10–19 years) [20].

Teenage mothers have poor birth outcomes such as low birth weight, preterm delivery, stillbirth, fetal and neonatal mortality, and severe neonatal problems [4,21–23]. Furthermore, these groups also have a higher chance of malnourishment, delayed physical and mental development, dispute between parents and children, and even inadequate academic achievement [4,24]. Furthermore, unmarried and pregnant teens faced societal repercussions that led to rejection, stigma, and abuse from peers, parents, and partners [25]. Similar to this, early pregnancy and child rearing cause girls to drop out of school, which affects their chances for future education and employment [26–28].

Prior studies have indicated that a crucial strategy for lowering the number of issues related to teenage contraceptive use is to offer comprehensive, high-quality sex and relationship education [29].

Adolescent pregnancy remains a serious public health concern in Ethiopia, despite efforts by governmental and non-governmental organizations to lower the rate of adolescent pregnancies. These efforts include passing legislation prohibiting early marriage, creating a national strategy for adolescent and youth reproductive health, legalizing abortion, creating a youth and HIV/AIDS policy, and organizing communities against harmful traditional practices, including linking such practices to the recently passed criminal code. Thus, the purpose of this study was to evaluate, using EDHS 2019, the spatial distribution of adolescent pregnancy and early motherhood in Ethiopia. To lower the number of teenage pregnancies in the nation, policymakers and program planners will benefit from the study findings as they develop and implement strategies and initiatives

## Method

### Study design and setting, and population and sampling procedures

The 2019 Ethiopia Demographics and Health Survey was used in this study. The Federal Democratic Republic of Ethiopia (FDRE) is a landlocked country in the Horn of Africa. Eritrea and Djibouti border it to the north, Somaliland to the northeast, Kenya to the south, South Sudan to the west, and Sudan to the northwest. Moreover, it contains a range of geographical features, with altitudes varying from 125 m below sea level in the Afar Depression to 4550 m above sea level in the Amhara Region ‘Ras Dejen’, Semien Mountains and Ethiopia, with a total area of 1,100,000 square kilometers (420,000 square miles) [30]. Additionally, it is the world’s 12th most populous country and Africa’s second most populous. Ethiopia is divided into eleven national regional states: Tigray, Afar, Amhara, Oromia, Somalia, Benishangul-Gumuz, Sidama, Southern Nations Nationalities and People Region (SNNPR), Gambella, and Harari, as well as two administrative states (Addis Ababa City administration and Dire Dawa City Council). According to estimates based on the latest United Nations data, the population of Ethiopia is currently 123,415,729 [31]. Moreover, the young population consists of 48,479,577, which is 39.80% of the whole population of Ethiopia [32]. In addition, reproductive health coverage in Ethiopia is low; however, it is rising as evidenced by three consecutive EDHS studies (i.e., 2000, 2005, and 2011)) [33,34]. A pillar of unwanted pregnancy prevention is the use of highly effective contraceptive methods. However, according to the 2016 Ethiopian Demographic and Health Survey (EDHS) contraceptive use remains low at 39.6% [35].

The source of the population was all pregnant and early motherhood (15–19 years old) among late adolescents in Ethiopia. The study population comprised pregnant and early motherhood girls who were pregnant and/or had a child within the previous 5 years of survey. Our study had a total sample size of 2210 pregnancies and early motherhood among late adolescents. Ethiopia has 11 geographical regions (nine regional states and two city administrations). Subsequently, the regions were divided into “zones” “woreda,” “kebeles,” and “kebele” and subdivided into census enumeration areas (EAs). EDHS samples were stratified and selected in two stages. Each region is stratified into urban and rural areas. In the first stage, based on the 2007 Ethiopian Population and Housing Census (PHC), EAs were selected with a probability proportional to their size. In EDHS 2019, 305 clusters were selected. In the second stage, a fixed number of households per cluster were selected from the newly produced household list using an equal probability systematic selection method. The number of households was 9150 in the EDHS year 2019.

**Fig 1:**
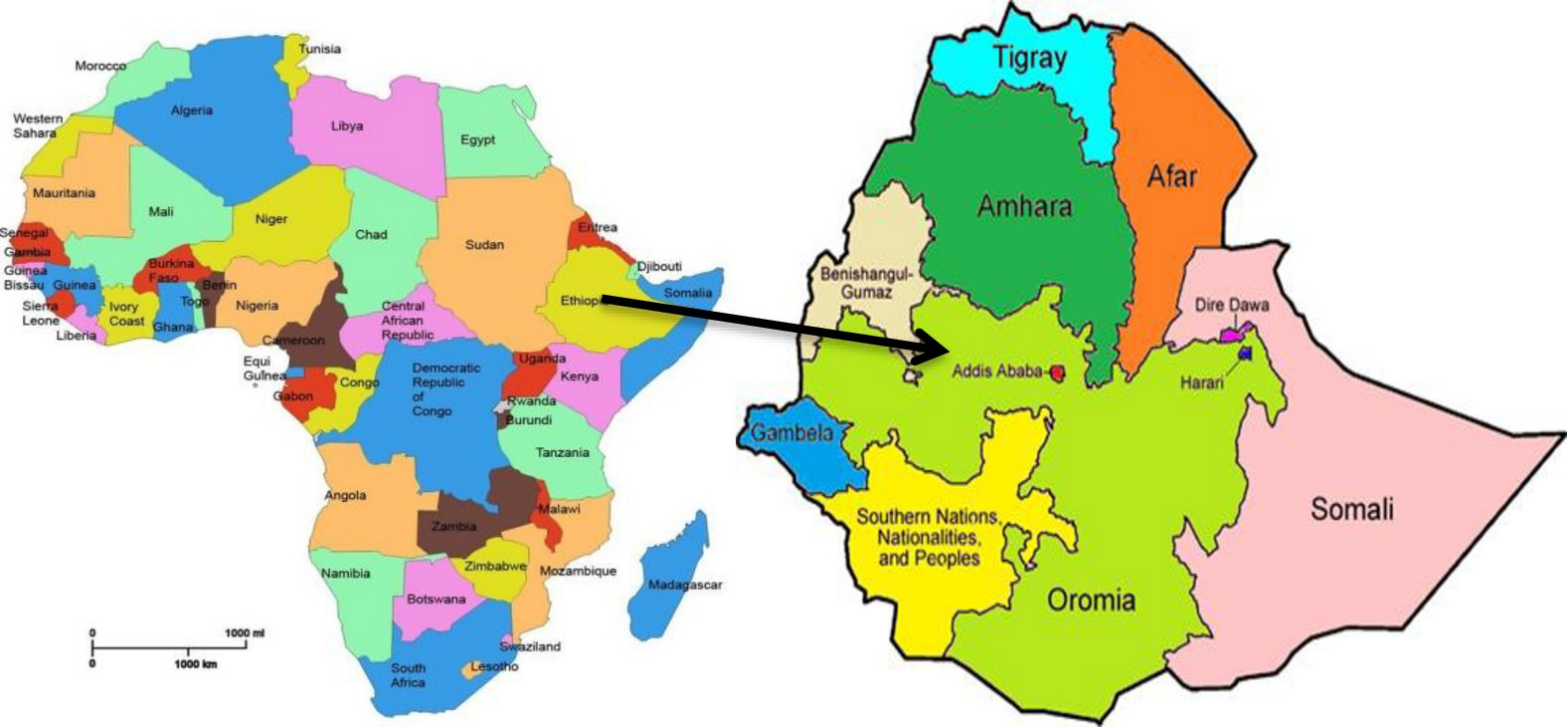
This picture depicts the Ethiopia map with its region.

Moreover, all DHS questionnaires were customized in the Ethiopian context by different experts. All questionnaires were developed in English and then translated into principal languages, such as “Amarigna,” “Tigrigna,” “Oromiffa,” “Somaligna,” and “Afarigna” languages. The quality of the EDHS data was maintained by checking all questionnaires before and after data entry [36] (***Fig* 1**).

### Study variable

#### Dependent variable

Late adolescents (15–19) who had a child or pregnancy in the past five years before survey were considered as a dependent variable. The response variable was dichotomized into "late adolescent pregnancy and early motherhood = 1" and "late adolescent non-pregnancy and early motherhood = 0".

#### Independent variables

There are various candidate individual level variables (model-I) included in the analysis model, such as age, educational attainment, marital status, religion, wealth index, contraceptive use and intention, and relationship to household head. The community level variables (model-II) are residence, region, community women’s education, and community poverty status.

#### Operational definitions

The World Health Organization defines late adolescence, which lasts from 15 to 19 years, as a phase of rapid growth in terms of physical, cognitive, and psychological development between childhood and adulthood [1]

Late-adolescent pregnancy: it is defined as the occurrence of pregnancy in girls aged 15–19 years [4]

**Wealth index:** according to the EDHS definition, households are graded on the basis of the number and type of consumer goods they own, which can range from a television to a bicycle or automobile, as well as dwelling qualities such as drinking water source, bathroom facilities, and flooring materials. Principal component analysis was used to calculate these scores.

**Community female education**: The overall sum of women’s educational attainment as determined by the median distribution of educational attainment in the community. If the proportion of women in the community with secondary education or higher was between 0% and 13.7%, it was considered low; if it was between 13.8% and 100%, it was considered high.

**Community-women’s poverty:** Using the same process, this variable is obtained from the wealth index of a particular household. In a given community, it was considered high if the proportion of women in the two quintiles of lowest wealth was 27.9%–100% and low if it was 0%–29%.

### Data management and statistical analysis

Data were extracted from the EDHS 2019 dataset, particularly individual record (IR) files. Data were cleaned, recoded, and weighted using STATA 14 statistical software before statistical analysis. Descriptive statistics and analytical analysis were performed using STATA version 14 statistical software and Microsoft Excel. ArcGIS version 10.7 was used to determines the geographic distribution of pregnancy and early motherhood in Ethiopia and create a representative map. Fixed effect estimates with a 95% confidence interval measure the association between individual and community-level factors and the odds of late adolescent pregnancy and early motherhood. A P-value of 0.05 was reported as a significant factor affecting adolescent pregnancy and early motherhood.

The Median Odds Ratio (MOR), which is the median value of the odds ratio between the area at the lowest risk and the highest risk when two clusters are randomly chosen, was used to assess the measure of variance. MOR = e0.95√VA or, MOR = exp. [√ (2 × VA) × 0.6745], where; VA is the area level variance. The Proportional Change in Variance (PCV reveals the variation in early pregnancy and motherhood exposure among late adolescent girls whose age is between 15–19 years old explained by factors. The PCV is calculated as = $\frac{Vnull-VA}{Vnull}$*100. Where: Vnull is the initial model’s variance and VA is the model’s variance with additional terms. Also, the Intra Class Correlation Coefficient (ICC), a measurement of the variation in pregnancy & early motherhood between clusters, is computed as; ICC = VA÷VA+3.29 *100%, where; VA = area/cluster level variance.

Intraclass correlation coefficient (ICC): ICC = (Between-group variance) / (Between-group variance + Within-group variance)

Proportional change in variance (PCV): PCV = [(Variance of the null model)—(Variance of the model with random effects)] / (Variance of the null model)

Median odds ratio (MOR): MOR = exp(0.5 * (π^2^ / 3)), where π is the mathematical constant pi (approximately 3.14159)

Deviance Information Criterion (DIC): DIC = -2 * (Log-likelihood) + (2 * Effective number of parameters), where the effective number of parameters is a measure of model complexity.

Log-likelihood ratio (LLR): LLR = -2 * (Log-likelihood of the null model—Log-likelihood of the model being compared)

We used four models to estimate the fixed effects of individual and community-level factors and the random intercept of between-cluster variation. These are the null model without predictors, model I with only individual-level variables, model II with only community-level variables, and model III with both individual-level and community-level variables.

### Spatial autocorrelation

Arc GIS 10.7 software was used for spatial autocorrelation and the detection of hot spot areas. Moran’s I is a popular spatial autocorrelation indicator. Global Moran’s I was used to measure spatial autocorrelation. It has a value between -1 and 1.”1” denotes perfect positive spatial autocorrelation (high or low values cluster together), “**-**1” denotes perfect negative spatial autocorrelation (a checkerboard pattern), and “0” denotes perfect spatial randomness [37].

### Hot spot analysis

To conduct a hot spot analysis, the prevalence of late adolescent pregnancy and early motherhood was computed for each enumeration. The output is then interpreted as a feature with a high z-score and low p-value, indicating spatial clustering of high values (hot spot). A low negative z-score and a small p-value indicate that low values (cold spots) are spatially clustered.

### Spatial interpolation

A spatial interpolation method was employed to predict the value of late adolescent pregnancy and early motherhood in unsampled locations, considering the sampled value in enumeration areas (EAs). It is a multi-step process that considers the degree of variation and the distance between familiar data points when calculating values for unknown locations. It assigns weights to nearby calculated values to forecast unmeasured locations using ArcGIS 10.7 software.

### Spatial scan statistics

The spatial scan statistic using Bernoulli’s model was employed to detect significant cluster windows of adolescent pregnancy and early motherhood. As a result, the number of cases in this study comprised adolescents who had a history of pregnancy and/or childbirth 5 years before the survey. Adolescents with no history of pregnancy or childbirth are controlled. In the entire study area, many scanning windows of various sizes are constructed across all locations. Each scanning window is a potential cluster candidate. Circular scanning windows are considered in SaT Scan’s maximum scanning window size (MSWS) and the area is set to 50% of the total population by default. When compared with areas outside the window, areas with a high log-likelihood ratio (LLR) and p = 0.05 were considered high-risk. Finally, significant and most likely clusters were identified using LLR, relative risk, and p value. The location where the most likely clusters was determined using ArcGIS V.10.6. Based on 999 Monte Carlo replications; the primary and secondary clusters were identified, given p values, and ranked based on their likelihood ratio test.

### Ethics approval and consent to participate

Demographic and Health Survey granted data access permission via an online request at http://www.dhsprogram.com. The data used in this study was freely available and did not contain any personal information. Our research was based on secondary data from the Ethiopian Demographic and Health Survey, and we received a letter of approval from the Demographics Health and Survey. There was no patient or public involvement in this study.

## Result and discussion

### Sociodemographics and economic characteristics of the participants

A total of 2210 adolescent girls between the age of 15 and 19 years were included in this analysis. More than one quarter (27.05%) of the adolescents was 18 years old. Slight half of the participants 1,132 (54%) attained incomplete primary education. Regarding residence, the majority (68.52%) of the participants resided in rural areas. Nearly three quarters (73.62%) of adolescents were never in union, and the majority (40.86%) of them was Muslim in religion. Regarding the wealth index, most participants 646 (30.76%were the richest. More than half of house heads 19.9%) had a relationship with their daughter. Regarding contraception use and intention, a significant number of them 1933 (92.05%) did not intend to contraceptive (**Table 1)**.

**Table 1:** Sociodemographics characteristics of late adolescents who had a pregnancy or children in the past five years preceding the survey in Ethiopia (n = 2210).

Variables	Category	Unweighted frequency (%)	Weighted frequency (%)
Age			
	15	478(22.76)	518(23.44)
	16	429(20.43)	455(20.60)
	17	333(15.86)	332(15.03)
	18	568(27.05)	620(28.04)
	19	292(13.90)	285(12.89)
Educational Attainment			
	No Education	304(14.48)	235(10.65)
	Incomplete Primary	1,132 (54.00)	1,258(56.92)
	Complete Primary	180(8.57)	205(9.26)
	Incomplete Secondary	385(18.33)	436(19.75)
	Complete Secondary	9 (0.43)	8(0.36)
	Higher	88 (4.19)	67(3.05)
Place of residence			
	Urban	661 (31.48)	740 (33.48)
	Rural	1 439(68.52)	1470 (66.52)
Marital status			
	Never In Union	1546 (73.62)	1,645(74.45)
	Married	441(21.00)	449(20.30)
	Living With a Partner	35(1.67)	26(1.20)
	Widowed	3 (0.14)	2(0.07)
	Divorced	52 (2.48)	56(2.54)
	Separated	23(1.10)	32(1.44)
Religion			
	Orthodox	793(37.76)	949(42.92)
	Catholic	17 (0.81)	14(0.65)
	Protestant	417 (19.86)	633(28.66)
	Muslim	858 (40.86)	594(26.88)
	Traditional	9 (0.43)	15(0.67)
	Other	6(0.29)	5(0.22)
Wealth Index			
	Poorest	464 (22.10)	322(14.55)
	Poorer	330 (15.71)	411(18.58)
	Middle	315(15.00)	447(20.21)
	Richer	345 (16.43)	497(22.50)
	Richest	646 (30.76)	534(24.16)
Contraceptive Use and Intention			
	Using the modern method	164 (7.81)	207(9.39)
	Using the traditional method	3 (0.14)	0.32(0.01)
	does not intend to use	1,933(92.05)	2,002(90.60)
Region			
	Tigray	167(7.95)	141(6.37)
	Afar	126 (6.00)	16 (0.74)
	Amhara	223 (10.62)	508(22.98)
	Oromia	276 (13.14)	879(39.78)
	Somali	173(8.24)	111(5.03)
	Benishangul	186 (8.86)	26(1.16)
	SNNPR	235(11.19)	404(18.27)
	Gambela	177(8.43)	9(0.42)
	Harari	174 (8.29)	6(0.29)
	Addis Ababa	172 (8.19)	94(4.26)
	Dire Dawa	191(9.10)	16(0.70)
Relationship with the household head			
	Head	75 (3.57)	80(3.60)
	Wife	251 (11.95)	274(12.42)
	Daughter	1, 258(59.90)	1383(62.58)
	Daughter-In-Law	33 (1.57)	49(2.20)
	Granddaughter	77 (3.67)	83(3.77)
	Sister	94 (4.48)	95(4.28)
	Other Relative	134(6.38)	140(6.33)
	Adopted/Foster Child	9 (0.43)	4(0.17)
	Not Related	115 (5.48)	66(3.01)
	Niece	54(2.57)	36(1.65)

### Prevalence of adolescent pregnancy and early motherhood in Ethiopia

**Fig 2:**
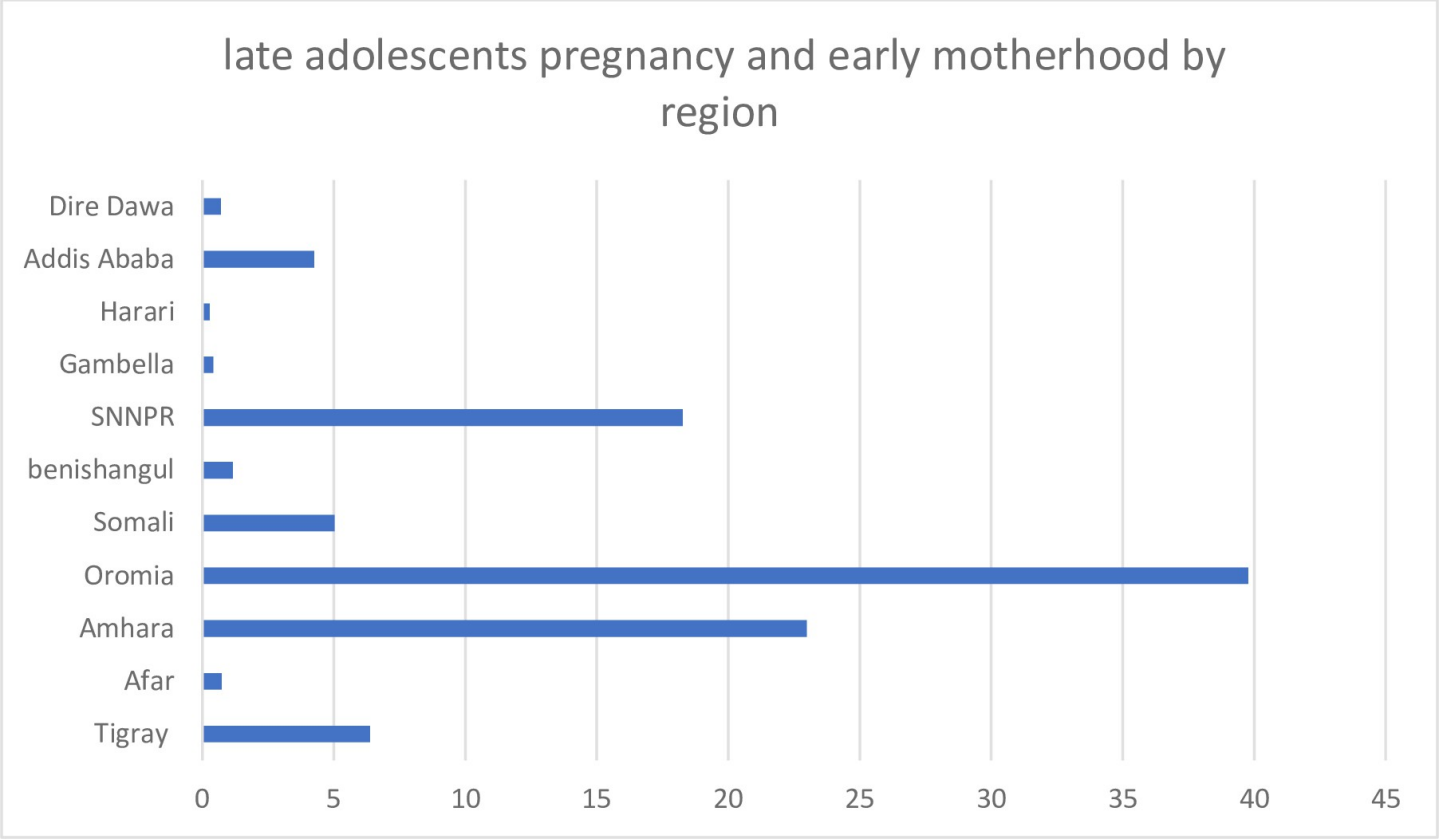
Prevalence of pregnancy and early motherhood among late-adolescents in each Ethiopia region in the 2019 EDHS year.

In Ethiopia (in 2019), the overall national prevalence of adolescent pregnancy and early motherhood was 12.89% (95%, CI, 11.56, 14.36). The prevalence was highest in the Oromia region (39.77% (95%, CI, 37.75, 41.83) and lowest in the Harari region (95%, CI, 0.28% (0.13, 0.62) (***Fig* 2**).

### Spatial analysis

#### Spatial autocorrelation (Global Moran’s I)

**Fig 3:**
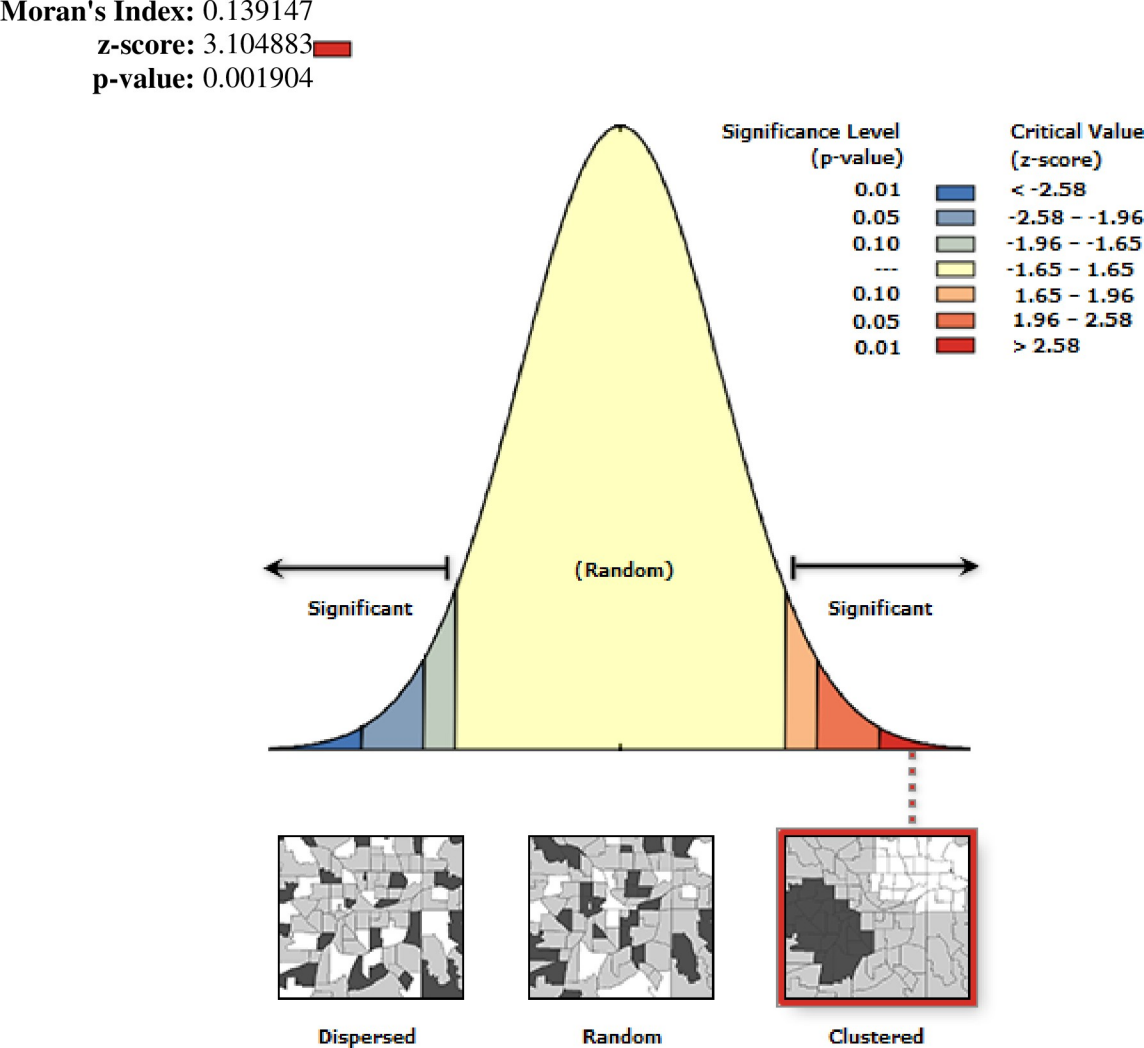
Global autocorrelation of pregnancy and early motherhood in Ethiopia, 2019.

The global spatial autocorrelation analysis showed that the spatial distribution of adolescent pregnancy and early motherhood varied across Ethiopia with a global Moran’s I value (I = 0.014, P<0.001), which indicates spatial clustering of adolescent pregnancy and early childhood (**Fig 3**).

#### Hotspot analysis (Getis-Ord Gi*)

**Fig 4:**
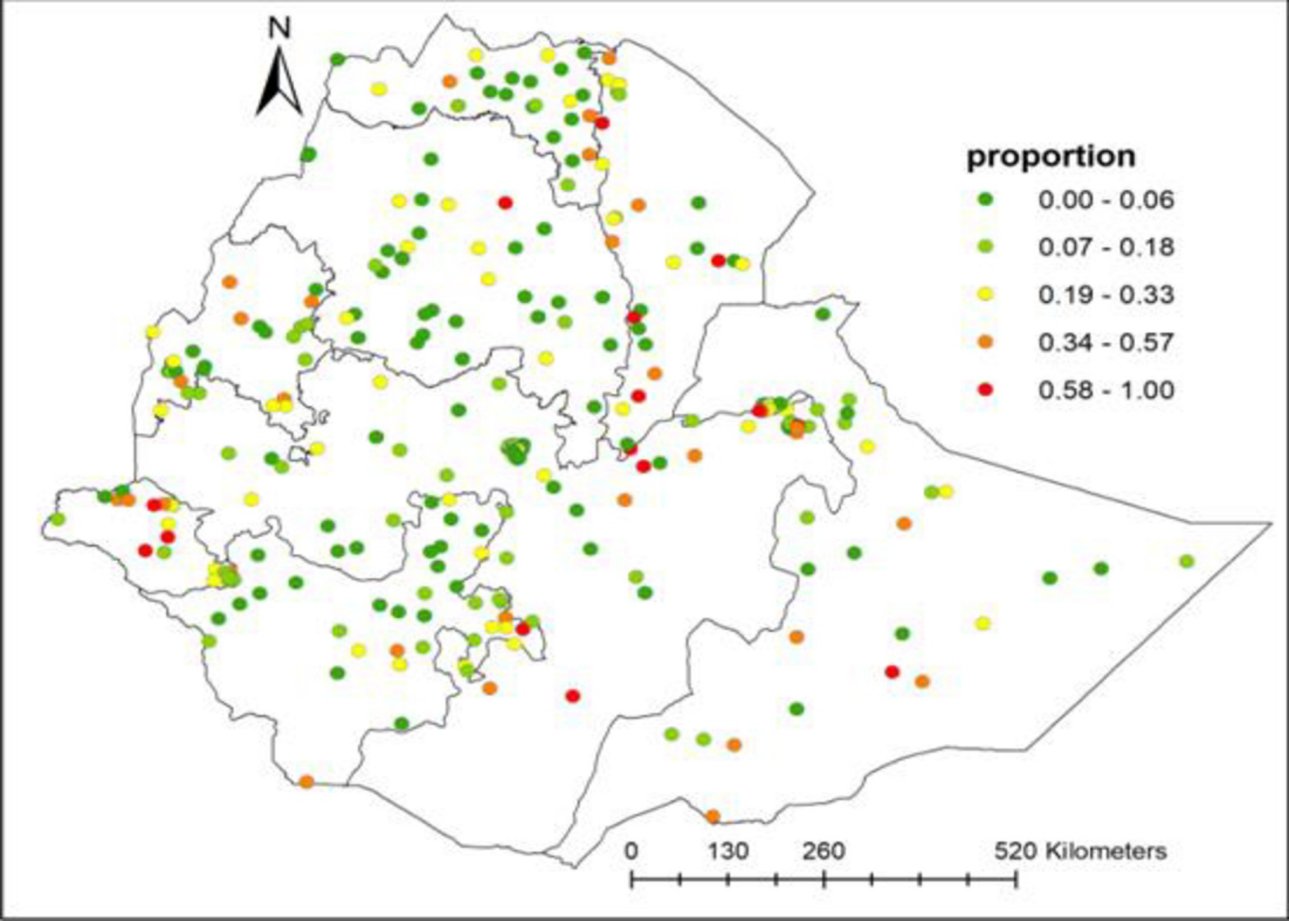
Spatial distribution of pregnancy and early motherhood in Ethiopia, 2019 (Source, CSA: 2013).

In the hotspot (Getis-Ord Gi*) analysis output, spatial clustering of adolescent pregnancy and early motherhood was observed in most parts of Gambella, Afar, ‎Benishangul-Gumuz, and the eastern part of the Oromia and Somali regions **(Table 2 and Fig 4**).

**Table 2:** Sat scan analysis results for pregnancy and early motherhood among late adolescents.

cluster	Enumeration areas(cluster detected)	Coordinates (radius)	population	cases	RR	LLR	P-value
1.	213, 212, 214, 206, 209, 208, 211, 207, 230, 229, 225, 217, 228, 218,226, 221	(7.813123 N, 34.536369 E) / 98.81 km	111	39	2.46	14.02	0.00033
2.	34, 30, 45, 31, 33, 26, 32, 29, 46, 44, 19, 24, 18, 36	(12.346083 N,41.005798 E) / 175.28 km	84	28	2.28	8.74	0.038
3.	305	(9.521566 N,41.739326 E) / 0 km	7	6	5.66	8.57	0.044
4.	142, 141, 136, 125, 138, 143, 137, 123, 144, 134, 145, 111, 135, 133,110, 114, 131, 103, 122, 117, 132, 183, 102, 113, 140, 106, 129, 186, 88, 89, 181, 250, 105, 248	(5.479641 N,42.196835 E) / 419.95 km	230	58	1.78	8.49	0.047
5.	254, 255	(9.327575 N,42.215199 E) / 3.80 km	12	7	3.85	5.83	0.365
6.	78	(12.345158 N,38.647789 E) / 0 km	8	5	4.11	4.59	0.737
7.	167, 168, 169	(9.684778 N,35.956673 E) / 18.63 km	30	11	2.43	4.12	0.852
8.	159, 160	(11.267438 N,35.292873 E) / 57.17 km	14	6	2.82	3.03	0.998

#### Spatial interpolation

**Fig 5:**
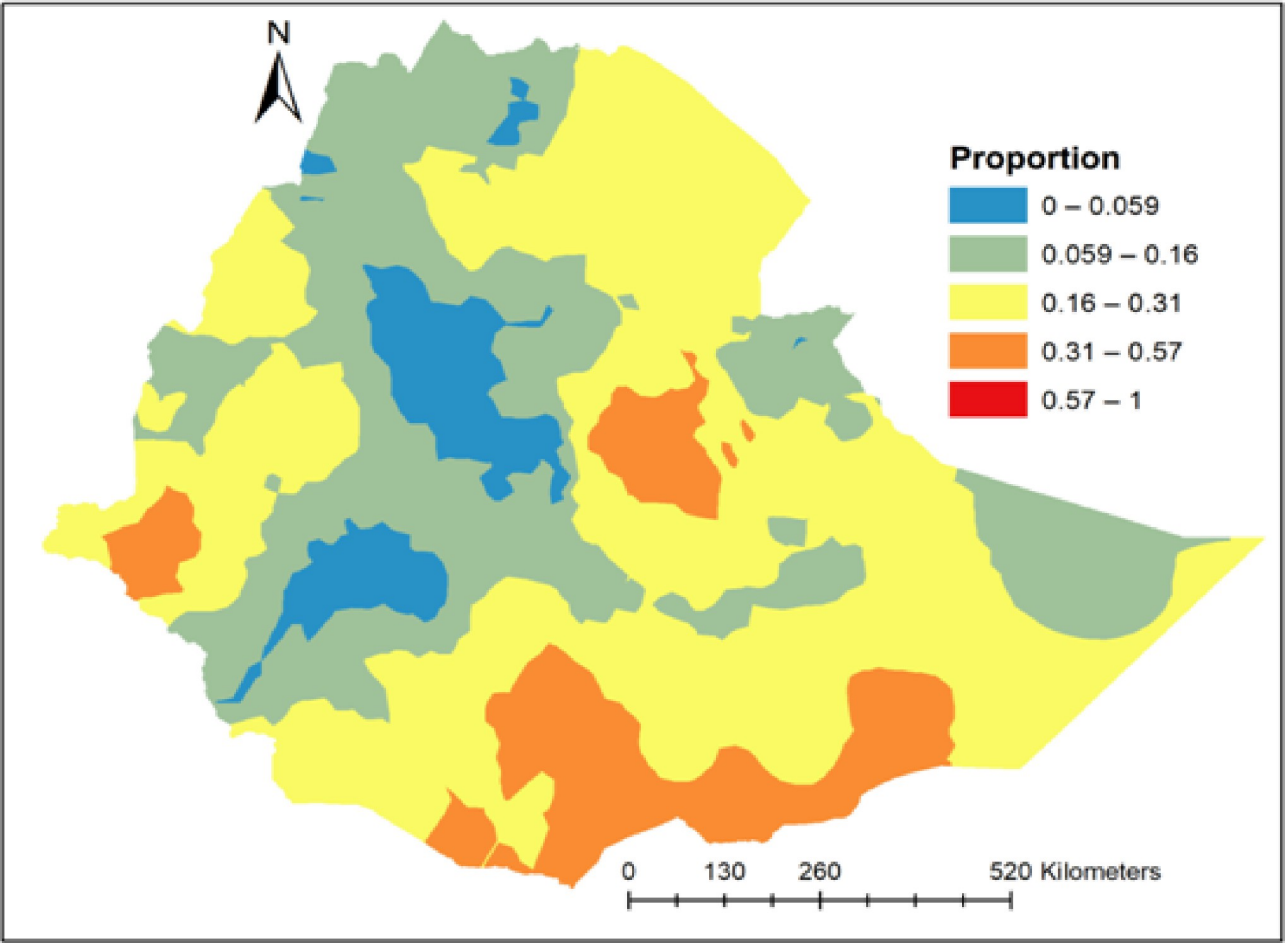
Kriging interpolation of pregnancy and early motherhood in Ethiopia, 2019 (Source, CSA: 2013).

*The Kriging interpolation technique generates a high prevalence prediction of late adolescent pregnancy and early motherhood*, *which was identified in some areas of Ethiopia*, *including the southern part of Somalia and Afar*, *the central Gambella*, *and the northeastern and southern parts of Oromia*. *In contrast*, *low prevalence prediction was observed in Addis Ababa*, *Central Tigray*, *SNNPR*, *Dire Dawa*, *eastern Somalia*, *Benishangul-Gumuz*, *and the Amhara region*. ***Fig 5***.

### Spatial scan statistical analysis

**Fig 6:**
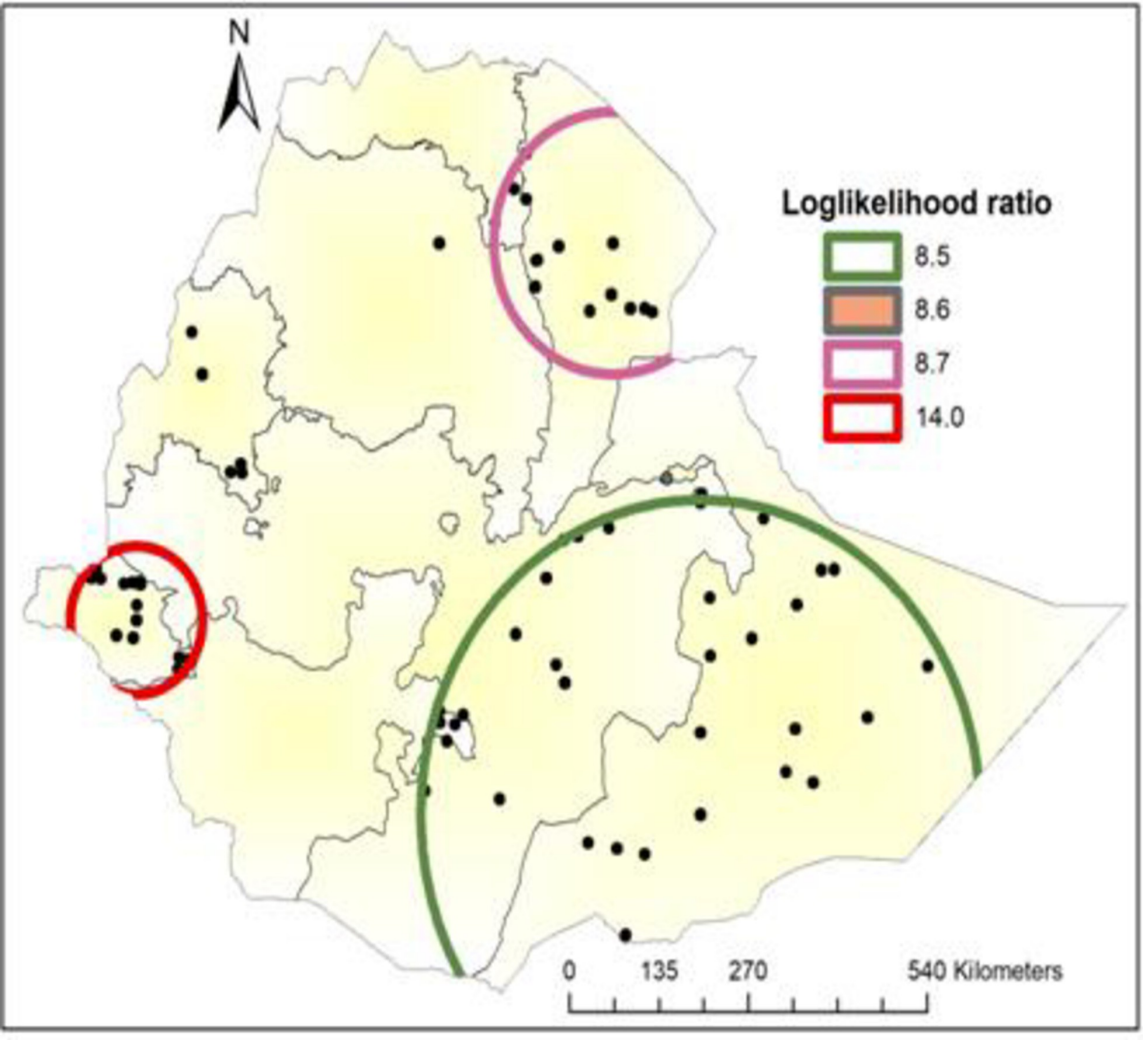
Sat Scan analysis of hotspot areas of pregnancy and early motherhood in Ethiopia, 2019 (Source, CSA: 2013).

*The spatial scan analysis demonstrated those 55 significant clusters*, *of which the primary*, *secondary*, *tertiary*, *and quaternary clusters consisted of 16 clusters*, *14 clusters*, *one cluster*, *and 24 clusters*, *respectively*. *The primary satellite scan window was detected in the Gambela region at 7*.*813123 N*, *34*.*536369 E with a radius of 98*.*81 km*. *This cluster window comprises 111 populations* and 39 cases with a relative risk of 2.46 and a log likelihood ratio of 14.022864 at a p-value of 0.00033. The late adolescents had a 2.46 times higher risk of developing an adolescent pregnancy and early motherhood during this scanning window than the late adolescents outside the window. The secondary cluster was also identified by SaT Scan as the most likely cluster and did not spatially overlap with the most likely cluster. The secondary SaT Scan circular window is dominantly observed in the Afar region of Ethiopia at 12.346083 N, 41.005798 E, a distance of 175.28 km. Additionally, this cluster has 84 *populations* and 28cases with a relative risk of 2.28, and 8.74 is the likelihood of it with a p-value of 0.038statstically interpreted as late adolescents inside the circular window, had a 2.28 times higher likelihood of having an adolescent pregnancy and early motherhood than adolescents who were outside the window. Similarly, the SaT scan window analysis demonstrated that the third cluster is located predominantly in the Oromia and Somalia regions at 9.521566 N and 41.739326 E with a radius of 0 km. It also has seven populations and six cases with a relative risk of 5.66 and a log-likelihood ratio of 8.57 with a p-value of 0. 044. The study population inside the circular window had a 5.66 times greater chance of becoming pregnant and early motherhood than the teenage population outside the spatial window. The Quaternary cluster is geostatistical at 5.479641 N, 42.196835 E and has a radius of 419.95 km, not overlapping spatially with other significant clusters. Two hundred and thirty *populations and fifty-eight* cases existed in the cluster with a relative risk of 1.78 and a log-likelihood ratio of 8.49, with a P-value of 0.047. Compared with adolescents outside the circular window, the adolescents inside the circular window had a 1.78 times probability of becoming pregnant and early motherhood ***Fig 6***.

### Individual and community-level factors associated with late-adolescent pregnancy and early motherhood in Ethiopia

#### Random effect analysis results

In the null model, the value of ICC was 84.6%, which implies that the total variability in pregnancy and early motherhood was a result of the difference between clusters, whereas the remaining 15.4% was attributable to individual differences. The combined individual a community fitted level model (model III) had the best-fitted model because it had the lowest deviance value in contrast to other models.

#### Fixed effect analysis results

Various variables were run in the multivariate multilevel logistic regression analysis, including age, marital status, religion, educational attainment, wealth index, contraceptive use and intention, relationship to household head, place of residence, region, community women’s education, and community poverty status. From these, the age of adolescents, marital status, educational attainment, and region were significantly associated with adolescent pregnancy and early motherhood.

The odds of being pregnant and early motherhood were 3.43(AOR = 3.43; 95% CI: 1.54–7.59),14.92 (AOR = 14.92; 95% CI: 6.78–32.8), 8.44(AOR = 8.44; 95% CI: 4.06,17.56) times more likely among the age group of 17 years, 18 years, and19 years, respectively, compared with the age group of 15 years. Regarding marital status, the probability of being pregnant and early motherhood was 25.38 times (AOR = 25.38; 95% CI: 15.33, 42.02) higher among married adolescents than their counterparts. Adolescents who had complete primary, complete secondary, and higher educational status were 2.22times (AOR = 0.45; 95% CI: 0.21–0.95) less likely to experience pregnancy and early motherhood than those who had no educational status. The likelihood of experiencing pregnancy and early motherhood was 3.64 times (AOR = 3.64; 95% CI: 1.04, 12.75) more likely to occur in the Gambela region of Ethiopia than in the counterparts (Table 3).

**Table 3:** Multivariable multilevel logistic regression analysis of individual and community-level factors associated with late-adolescent pregnancy and early motherhood in Ethiopia.

Individual and community-level variables	Models			
	Null model AOR (95%CI)	Model I AOR (95%CI)	Model II AOR (95%CI)	Model III AOR (95%CI)
Age				
15		1		
16		2.21(1.01,4.80)		
17		7.64(3.75,15.58)		
18		19.69(10.16,38.1)		
19		34.25(17.03,68.9)		
Marital status				
Married		48.94(33.10,72.37)		
Others		1		
Religion				
Orthodox		1		
Muslim		2.12(1.40, 3.22)		
Others***		1.92(1.34, 2.75)		
Education attainment				
No formal education		1		
Incomplete primary		0.49(0.35,0.70)		
Incomplete secondary		0.29(0.18,0.47)		
Others**		0.33(0.20,0.56)		
Wealth index				
poorest		1		
Poorer		1.09(0.73,1.62)		
middle		0.89(0.58,1.36)		
richer		0.57(0.37, 0.89)		
Richest		0.34(0.22,0.52)		
contraceptive use and intention				
Modern contraceptive		7.04(4.67, 10.62)		
Others*		1		
Relationship to household head				
Wife		33.67(22.15,51.1)		
daughter		1		
Others****		3.12(2.17,4.46)		
Place of residence				
Urban			1	1
Rural			2.60 (1.75,3.86)	0.88(0.47, 1.66)
Region				
Tigray			4.40(1.54,12.63)	1.18(0.31,4.37)
Afar			12.79(4.52,6.11)	2.06(0.54,7.84)
Amhara			2.37 (0.82,6.84)	0.46(0.12,1.75)
Oromia			5.16(1.91,13.95)	1.07(0.31,3.69)
Somali			7.19(2.57, 20.12)	1.23(0.32,4.59)
Benishangul			7.20(2.59,19.98)	1.47(0.40,5.37)
SNNPR			4.30(1.56,11.85)	0.73(0.20, 2.70)
Gambela			10.47(3.80,8.84)	3.64(1.04,12.75)
Harari			4.84(1.69, 13.78)	1.69(0.48,5.92)
Addis Ababa			1	1
Dire Dawa			5.27(1.87,14.86)	1.51(0.45, 5.11)
Community women’s education				
Lower community education			1	1
Higher community education			1.35(0.95,1.91)	1.63(0.70,3.81)
Community poverty status				
Lower community poverty			1.986(1.41,2.79)	1.92(0.81,4.55)
Higher community poverty			1	1
Random effects and model comparison				
ICC	20.46(13.72,29.4)	14.08(6.19,28.9)	11.06(3.03,22.91)	10.04(2.01,21.89)
PCV (%)	Reference	43.7	58.01	61.27
MOR	3.12(2.83,3.46)	2.35 (1.71, 4.08)	2.11(1.95,2.30)	2.05(1.49, 3.88)
Deviance	1,758.73364	979.82862	1,679.6913	950.21276
Log-likelihood	-879.36682	-489.91431	-839.84565	-479.10638

**Note:** *ICC*: *Intraclass correlation Coefficient; AOR*: *Adjusted Odds Ratio; SNNPR*: *Southern Nation*, *Nationalities*, *& people’s region*, *others*

*: using the traditional method and does not intend to use, others

**: Complete primary, complete secondary, and higher education, Others

***: never in a union, living with a partner, widowed, divorced and no longer living together/separated, Others

****: Head, son/daughter-in-law, grandchild, brother/sister, other relatives, adopted/foster child, not related, niece/nephew by blood.

Even though the rate of adolescent pregnancy has been steadily declining over the last decade, it remains a major public health concern with long-term consequences for teenage mothers, their infants, and families, as well as society as a whole. Therefore, effective strategies for preventing adolescent pregnancy include community programs to improve social development, responsible sexual behavior education, and improved contraceptive counseling and delivery [38].

The finding of our study showed that the prevalence of pregnancy and early motherhood among late adolescents was 12.89% (95%, CI, 11.56, 14.36). This finding is comparable with previous studies done in Arba Minch (7.7%), South Africa (11.0%), and EDHS 2016 (13%) [10,39,40]. This is might be due to similar demographic and socio- economic characteristics, and culture of early marriage, however, this finding is lower than studies done in Africa (18.8%), Latin America (19.1%), Nigeria (22.9%), ‘Farta Woreda’ (25.4%), Canada (27.6%), Pakistan (42.5%), Nigeria (49%), and East Africa (54.6%) [41–48]. This variation may be due to the fact that the magnitude of abortion was found to be significantly high in Ethiopia [49,50] because several women used abortion as a reducing of family planning [51] which contributes to reducing the prevalence of teenage pregnancy. Another possible reason might be due to differences in the number of adolescents involved in the study, and the geographical distribution of teenagers. In advanced countries, most late adolescents are victims of pornography movie exposure which has a significant association with premarital teenage pregnancy [52].

The SaT Scan analysis demonstrated two statistically significant spatial circular windows with high pregnancy and early motherhood. Similarly, the hot spot location of pregnancy and early motherhood were detected in Ethiopia, particularly at Gambella, Afar, ‎Benishangul-Gumuz, and the eastern part of Oromia, Somalia. A possible explanation could be that these countries are dominated by pastoral or nomadic regions and that the population lives in border areas, making it difficult to access health services. In addition, these regions are underdeveloped areas in Ethiopia with low health facilities coverage. These areas are also the hotspots of the highest poverty, which may be responsible for the fall of adolescents to pregnancy as a reason for adolescents engaging in various sexual activities to gain money. This finding is supported by qualitative research conducted in South Africa, which found that poverty has a significant impact on teenagers’ pregnancy [53], Whereas the cold spot areas of pregnancy and early motherhood were located in Tigray, Amhara, SNNPR, Oromia region of Ethiopia. One of the reasons could be that the highest modern contraceptive utilization in contrast to other parts of Ethiopia, such as the Amhara region accounting for 50%, and Addis Ababa for 48%%. Another important explanation is the fact that evidence of modern contraceptive use increases with increasing household wealth, from 27% among women in the lowest quintile to 51% among those in the highest quintile. Similarly, these countries have a higher literacy rate than other areas of Ethiopia. This is supported by the findings of the EDHS 2019 that the percentage of women using modern methods is higher in women with secondary education compared to women without education.

Our research shows that age, education, region, and marital status are determinants of teenage pregnancy and early motherhood. Accordingly, Teenagers aged 17 and up had a higher risk of teenage pregnancy and early motherhood than those aged 15 and under. Similar findings were reported in studies conducted in Wogedi, Tigray Eastern Ethiopia, Ethiopia, and Nigeria [41,48,54–56]. This may be because adolescents go through a process of sexual maturation, which includes physical changes as they get older, leading to an interest in and desire to learn about sexuality. Another explanation is that if the availability and accessibility of content containing pornography across various media continues to increase, young people will be more likely to encounter sexual content. Exposure to pornography at a young age can affect their attitudes and behaviors towards sex. In addition, young adults will be more likely to have sex because of peer pressure, a sense of freedom, and a romantic relationship, and they may be more vulnerable to sexual violence due to many factors, including greater exposure to more social environments and greater risk-taking behaviors [57].

In terms of marital status, married adolescents are more likely to become pregnant and give birth prematurely than widowed, living with their partner, divorced, separated, and not living together. This is in line with the finding from studies in Ethiopia [56], East Africa [55], Wagedi [58], Tigray [54], Nigeria [48], and Africa [43]. One explanation for this is the unmet need for contraception among adolescents in low- and middle-income countries. This suggests that they may want to prevent or delay pregnancy but may face problems accessing contraceptive services [59]. One of the problems identified in accessing protective services is the perceived lack of awareness regarding these services. This means that young women may be unsure about the availability, benefits, and proper use of birth control. This lack of information may lead to a higher risk of unintended and early pregnancy in this group [60].

Current findings show that adolescents with primary, secondary, and higher education are more than two times less likely to become pregnant and give birth earlier than uneducated youth. This finding is similar to studies conducted in Ethiopia [56], Africa [43], Eastern Ethiopia [55], Pakistan [44], Sub Sahara Africa [11], and East African countries [61]. This may be because educated adolescents have better knowledge and skills to prevent pregnancy. This is also supported by EDHS 2019 findings, which show that women with secondary education are more likely to use modern technology (56%) than uneducated women (32%). Additionally, girls who attend school for ten years will marry after six years because education increases independence, decision-making, and financial independence, and delays marriage. Each additional year of education is associated with a 10% decrease in pregnancy and a 10% increase in contraceptive use [62]. Moreover, teenagers who live in the Gambella region were nearly four times more likely to experience pregnancy and early motherhood compared to Addis Ababa. This might be because the probability of contraceptive utilization was 78 percent less likely utilized pastoralist communities in contrast to another area of Ethiopia **[63]** and the practice of early marriage is common in this region and thus it increases the risk of early motherhood, poverty, and termination of education.

### The strength and limitations of this study

We used weighting as a correction technique or statistical adjustment to the EDHS data to correct selection compensates occurred during the sampling period and compensate nonresponse rate of the research. Besides this, multilevel modeling or hierarchical linear models are also used to take the comparative advantage of nested data; however, the EDHS data has no clinical data, parents’ communication with their teenagers about a reproductive health issue, unavailable data such as the age of menarche, and early marriage.

## Conclusion

Overall, Late-adolescent pregnancy and early motherhood in Ethiopia are high as compared with the targets of the national adolescent and youth strategy [64]. At the individual level marital status, educational attainment, and age of adolescents were significant predictors for late pregnancy, and early motherhood, and regions were found as a community level associated with pregnancy and early motherhood among late adolescents. Our study also detect a spatiotemporal clusters of pregnancy and early motherhood among late adolescents in Gambella, Afar, ‎Benishangul-Gumuz, and the eastern part of Oromia, Somalia Therefore, Government ought to strengthen the implementation of the existed laws on reproductive rights of teenage girls and prevention of early marriage practice. To the ministry of education, better to design policies retaining pregnant girls in school, promoting, sex education particularly abstinence of sex, and adolescent girls better to get an education, about menstruation, sexual intercourse pregnancy and contraceptives before they reach to early adolescents.

## Supporting information

S1 Dataset (DTA)

## Abbreviations

AOR: Adjusted Odds Ratio
CI: Confidence Interval
CSA: Central Statistics Agency
EAs: Enumeration Areas
EDHS: Ethiopia Demographic Health Survey
GMI: Global Moran’s Index
ICC: Intraclass correlation Coefficient
LLR: *log Likelihood Ratio*
LMICs: income low- and middle-income countries
MSWS: Maximum Scanning Window Size
PHC: Population and Housing census
SNNPR: Southern Nation, Nationalities, & people’s region
